# Sarcopenia and associated factors according to the EWGSOP2 criteria in older people living in nursing homes: a cross-sectional study

**DOI:** 10.1186/s12877-022-02827-9

**Published:** 2022-04-21

**Authors:** Anna Escribà-Salvans, Javier Jerez-Roig, Miriam Molas-Tuneu, Pau Farrés-Godayol, Pau Moreno-Martin, Ester Goutan-Roura, Helena Güell-Masramon, Jordi Amblàs-Novellas, Dyego Leandro Bezerra de Souza, Dawn A. Skelton, Miriam Torres-Moreno, Eduard Minobes-Molina

**Affiliations:** 1 Research group on Methodology, Methods, Models and Outcomes of Health and Social Sciences (M3O), Faculty of Health Sciences and Welfare, Centre for Health and Social Care Research (CESS), University of Vic-Central University of Catalonia (UVic-UCC), C. Sagrada Família, 7, 08500 Barcelona, VIC Spain; 2 Research group on Tissue Repair and Regeneration Laboratory (TR2Lab), Faculty of Health Sciences and Welfare, Centre for Health and Social Care Research (CESS), University of Vic-Central University of Catalonia (UVic-UCC), C. Sagrada Família, 7, 08500 Barcelona, VIC, Spain; 3 Faculty of Health Sciences and Welfare, Centre for Health and Social Care Research (CESS), University of Vic-Central University of Catalonia (UVIC-UCC), C. Sagrada Família, 7, 08500 Barcelona, VIC, Spain; 4 Central Catalonia Chronicity Research Group (C3RG), Centre for Health and Social Care Research (CESS), Faculty of Medicine, University of Vic-Central University of Catalonia (UVIC-UCC), C. Sagrada Família, 7, VIC 08500 Barcelona, Spain; 5 Department of Collective Health, Federal University of Rio Grande do Norte, Natal, Rio Grande do Norte Brazil; 6 Research Centre for Health (ReaCH), School of Health and Life Sciences, Glasgow Caledonian University, Glasgow, UK

**Keywords:** Sarcopenia, EWGSOP2, Nursing homes, Dependence, Older adults

## Abstract

**Background:**

In 2018, the European Working Group on Sarcopenia in Older People (EWGSOP2) updated the original definition of sarcopenia, establishing new criteria to be used globally. Early diagnosis of sarcopenia in nursing home residents and the identification of contributing factors would target interventions to reduce the incidence of malnutrition, social isolation, functional decline, hospitalization and mortality.

**Aim:**

Verify the prevalence and the degree of severity of sarcopenia according to the new EWSGOP2 criteria and to analyse its associated factors in residents living in nursing homes in Central Catalonia (Spain).

**Design:**

A cross-sectional multicenter study was conducted in 4 nursing homes. SARC-F test was applied as the initial screening, muscle strength was measured by a dynamometer, skeletal muscle mass by bioimpedance analysis and physical performance by Gait Speed. Four categories were used: total probable sarcopenia, probable sarcopenia, confirmed sarcopenia and severe sarcopenia.

**Results:**

Among the total sample of 104 nursing home residents (mean age 84.6, ± 7.8; median 86, IQR 110), 84.6% were women and 85 (81.7%) (95% confidence interval [CI] 73.0-88.0) had total probable sarcopenia, 63 (60.5%) had probable sarcopenia, 19 (18.3%) had confirmed sarcopenia and 7 (6.7%) had severe sarcopenia. In the bivariate analysis, obesity was negatively associated and total time in sedentary behavior positively associated with all sarcopenia categories. In addition, malnutrition and urinary continence were positively associated with total and probable sarcopenia. Urinary incontinence was a positive associated factor of total and probable sarcopenia. In the multivariate analysis, obesity represented a negative associated factor: OR = 0.13 (0.03 - 0.57), *p* = 0.007 and OR = 0.14 (0.03 - 0.60), *p* = 0.008 with total and probable sarcopenia, respectively, adjusted by urinary incontinence status. For confirmed sarcopenia, obesity also represented a negative associated factor OR = 0.06 (0.01 - 0.99), *p* = 0.049 and the total time in sedentary behavior a positive associated factor OR = 1.10 (1.00- 1.20), *p* = 0.040.

**Conclusions:**

According the EWGSOP2 criteria, high prevalence of sarcopenia was found in institutionalized older people, ranging from 6.7 to 81.7% depending on the category. Malnutrition, urinary incontinence and total time in sedentary behavior were associated with sarcopenia, whilst obesity represented a protective factor in this population.

## Introduction

Sarcopenia is a pathology related to the loss of strength and muscle mass in older people [1]. This loss of muscle mass is associated with age, it affects the strength and functioning of older people and causes alterations at the bio psychosocial level [2]. In addition it leads to negative consequences such as falls, fractures, social isolation, functional decline, hospitalization and mortality [3]. Sarcopenia is thought to be prevalent in older adults, especially in those who live in nursing homes (NH) [4]. A recent meta-analysis has shown a high prevalence of sarcopenia in NH residents, ranging from 22 to 85%; this wide range is attributed to different diagnostic criteria [5].

In 2018, the European Working Group on Sarcopenia in Older People 2 (EWGSOP2) updated the original definition of sarcopenia to reflect new scientific and clinical evidence. The EWGSOP2’s updated recommendations aim to increase awareness of sarcopenia and its risk’s to health outcomes [6]. Preventative actions, such as exercise, can then be promoted [7]. The new definition incorporates the following aspects: low muscle strength as the first key determinant of diagnosis, new cut-off levels for the variables used to identify and characterize sarcopenia, and using the SARC-F questionnaire, or when clinically suspected to assess sarcopenia-associated symptoms, to identify individuals at risk of developing sarcopenia [6, 8]. The SARC-F questionnaire is a rapid diagnostic test for sarcopenia, with 5 components: strength, walking assistance, getting up from a chair, climbing stairs and falls [9].

Older adults who live in a NH are the frailest of our society, with high levels of functional limitations, physical dependence or cognitive impairment [10, 11]. Sarcopenia is highly prevalent in older NH residents but the prevalence varies considerably depending to the different profile of the population studied and with the different methods used to assess sarcopenia [5]. Early diagnosis of sarcopenia in NH residents would allow preventative actions to reduce the incidence of malnutrition, social isolation, functional decline, hospitalization and mortality [5].

The EWGSOP2 algorithm has already been applied to older people living in NHs [12] but there is still a gap in the identification of sarcopenia’s associated factors [5, 13]. Knowing these factors would allow practitioners to target early interventions for preventing, delaying, treating, and sometimes even reversing sarcopenia. This is relevant, because the prevalence of sarcopenia in Europe is likely to rise by 63.8–72.4% by 2045 [14]. Therefore, the main aim of this study is to verify the prevalence and the degree of severity of sarcopenia using the new EWSGOP2 criteria and analyse its associated risk factors in NH residents.

## Methodology

### Study design and population

A cross-sectional study was conducted from January to March 2020. Recruitment stopped because of the restrictions in Spain implemented due to the COVID-19 pandemic. The study follows the STROBE (STrengthening the Reporting of OBservational studies in Epidemiology) standards for cross-sectional studies [15]. The study was carried out in 4 NH in Osona (a region of Central Catalonia, Spain) and it is part of the OsoNaH project [10], registered in Clinical Trials (NCT04297904)*.*

All residents aged 65 years or over, permanently living in NHs were included. Subjects in a coma or palliative care (short-term prognosis) and those who refused to participate in the study were excluded. Those participants with severe cognitive impairment who could not follow the therapists’ instructions were excluded from the physical tests but included otherwise [16].

### Sample size

The sample size calculation was based on the study by Rodríguez-Rejón et al. (2019) [17] which also used the new EWGSOP2 criteria in NH residents. They found a prevalence of 60.1 and 58.1% of confirmed and severe sarcopenia, respectively, so 92 and 94 participants are necessary, considering an error factor of 10% [18].

### Consent and ethical approval

Ethical permission was obtained by the Ethics and Research Committee of the University of Vic - Central University of Catalonia (registration number 92/2019). Signed informed consent was gained from the resident or his/her legal guardian. All methods were performed in accordance with the relevant guidelines and regulations.

### Study procedures and data collection

Sarcopenia (main variable) was assessed according to EWGSOP2 criteria. The SARC-F questionnaire was used both to determine risk of developing sarcopenia and to assess the prevalence of sarcopenia. In order to confirm diagnosis and determine severity the following physical tests were assessed:

*Hand-grip muscle strength*, assessed using JAMAR Plus Digital Hand dynamometer [17, 18]. The resident held the dynamometer in their hand, with the arm at a right angle and the elbow at the side of the body. Two maximal strength hand grips were obtained from both hands. The highest value from the dominant hand was used for analysis. The reliability of measuring handgrip strength with the Jamar dynamometer is high (ICC ¼ 0.94; *p* < .001) in a clinically compromised population of geriatric patients [19].

The *amount of muscle* was measured with a Tanita TBF-300 bioimpedance device (Tanita Institute, Tokyo, Japan). The residents stood on the platform of the bioimpedance device and had to maintain the standing position without support for a few seconds. Bioimpedance analysis (BIA) is the validated tool for measuring muscle mass in adults [19, 20]. BIA equipment does not measure muscle mass directly, but instead derives an estimate of muscle mass based on whole-body electrical conductivity. Through the bioelectrical resistance (R), the skeletal muscle mass (SMM) was calculated using the formula in Jansen et al. [21]:

$$\mathrm{SMM}\ \left(\mathrm{kg}\right)=\left[\left({\mathrm{Ht}}^2/{\mathrm{R}}^{\ast}\times 0.401\right)+\left(\mathrm{gender}\times 3.825\right)+\left(\mathrm{age}\times -0.071\right)\right]+5.102$$

*Ht is height in centimetres; R is BIA resistance in ohms. For gender, men = 1 and women = 0. Age is in years.

Finally, *physical performance* was assessed using Gait Speed [22] from the Short Physical Performance Battery (SPPB) test. The individual is instructed to walk at a normal pace for 4 m, including acceleration and deceleration distance, twice, with the use of a walking aid if necessary, and the test is timed. The gait is timed and the result is recorded. Gait speed of longer than 5 s to walk 4 m (< 0.8 m/s) suggests an increased risk of frailty and the need for further clinical review [23]. Martinez BP et al. 2016, demonstrated that Gait Speed was a valid test with good reproducibility of physical performance in institutionalized older people (ICC = 0.99; *p* = 0.001) [23].

Participants with a final score of 4 or higher in SARC-F, were considered to be at sarcopenia risk. Regarding the physical tests, those individuals with sarcopenia risk and low muscle strength (< 27 kg for men and < 16 kg for women) were categorized as probable sarcopenia. Those individuals who had probable sarcopenia and low muscle quantity (< 20 kg for men and < 15 kg for women) were reported as confirmed sarcopenia. In that latter cases, measures of low physical performance (≤0.8 m/s) were used to categorize severe sarcopenia [6, 9, 22]. The category of total probable sarcopenia was composed by those subjects with probable sarcopenia and those with severe cognitive impairment, unable to perform physical tests and considered directly with low muscle strength [1]. Participants with mild and moderate cognitive impairment, followed the EGWSOP2 algorithm.

Sociodemographic information such as age, sex, the type of NH, chronic diseases, smoking and drinking habits, were obtained from the NH registers and checked with the NH professionals. Anthropometric variables (such as BMI, weight, and height) were measured using a Seca 213 measuring device, the Tanita TBF-300. The total number of medications in daily use were recorded, along with the types of medications, according to the *Anatomical Therapeutic Chemical* classification system [24]. Nutritional status was assessed using the Mini Nutritional Assessment (MNA) test [25]. Continence status was reported using Section H of Minimum Data Set (MDS) version 3.0 [26]. Functional capacity was measured using the modified Barthel Index, excluding continence items [27, 28]  Cognitive status was assessed using the Pfeiffer Scale [29]. Physical capacity was examined using the SPPB, including Gait Speed [30, 31]  Sedentary behaviour was assessed with the placement of the activPAL3TM activity monitor (PAL Technologies Ltd., Glasgow, UK) at mid-thigh. The device captured data continuously during both awake and sleep time, for 7 consecutive days [32, 33] . The following variables were extracted: number of steps in a day, duration in minutes of SB periods, total time in SB (%), SB bouts, total time in standing position and walking in hours, and transitions from sitting to standing over a 24 h period.

The approximate time to complete the physical tests and questionnaires with each resident was 30 to 45 min. The research team that collected the data was trained on the use of all tools and tests. The team collecting data were assessed for reliability of the handgrip dynamometer, the SPPB (including Gait Speed), BIA and anthropometric measurements, with calculation of the Kappa index and the interclass correlation coefficient (ICC) of the data from 20 residents. The ICC results were higher than 0.75 in all physical tests. The results from these 20 residents were included in the total final sample of the study.

### Statistical analysis

Descriptive analysis was undertaken, indicating absolute and relative frequencies for categorical variables. The dependent variables of the study correspond to the four categories of sarcopenia: total probable sarcopenia, probable sarcopenia, confirmed sarcopenia and severe sarcopenia. For these categories, prevalence was calculated with its confidence intervals at 95%. Bivariate and multivariate analysis was performed for these four dependent variables (Tables 2, 3, 4 and 5) [6]. The bivariate analysis was applied through the Chi-square test (or Fisher’s, when necessary) and the linear Chi-square test in case of dichotomous and ordinal variables, respectively. The Student T-test (or non-parametric Mann Whitney test) was used for quantitative variables. As an association measure, the Odds Ratio (OR) was calculated, with a confidence level of 95%. Multivariate analysis was performed by logistic regression with robust variance. All variables with a *p*-value ≤0.20 were tested for the multivariate analysis following the forward method. The adjustment of the final model was tested with the Hosmer Lemeshow test. A *p*-value < 0.05 was considered as statistically significant. Data were analyzed with SPSS version 27 (SPSS Inc., Chicago IL).

## Results

**Fig. 1:**
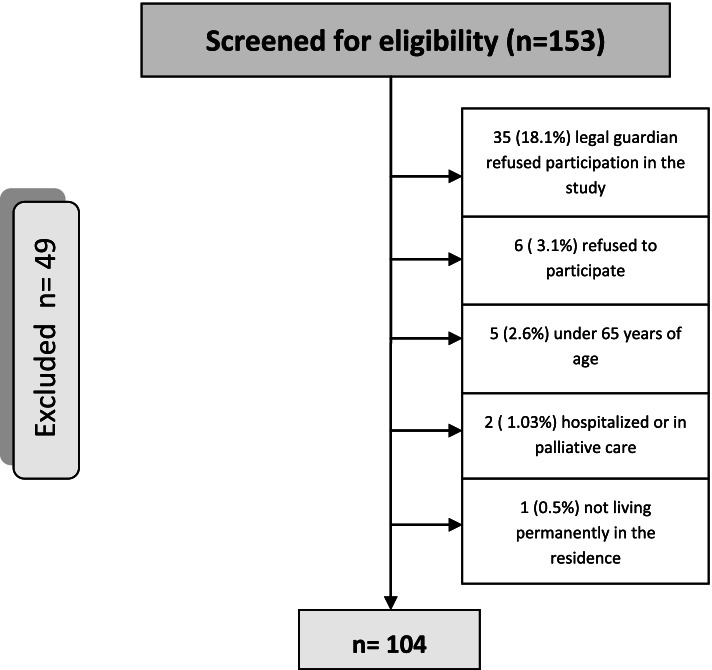
Flow chart of the sampling process.

We recruited 104 residents, representing 68% of the total residents in those NHs before we had to stop recruitment because of lack of access to the NHs in the COVID-19 pandemic (Fig. 1). Reasons for not being included in the study included both guardian and individual refusal to take part and a few not meeting age or residence criteria (Fig. 1).

The mean age of the participants was 84.6 years (SD = 7.8, median 86, IQR 11) and 88 [84.6%] were women. Six (3.1%) residents were smokers and 9 (4.7%) were alcohol drinkers. In total, 86 (82.7%) lived in state subsidized NHs and 18 (17.3%) in private NHs.

The mean number of chronic diseases reported was 5.0 [SD = 2.4 (median 5, IQR 3)]: 62 (32.5%) had hypertension, 62 (32.5%) dementia, 43 (22.5%) cardiac pathology, 32 (16.8%) depression, 32 (16.8%) diabetes mellitus II, 26 (13.6%) kidney failure, 21 (11.0%) cerebral stroke, 20 (10.5%) pulmonary pathology, 20 (10.5%) mental pathology, 18 (9.6%) cancer and 14 (7.3%) Parkinson’s Disease. The mean number of medications taken per day was 6.9 [SD = 3.8 (median 6.5, IRQ 5)].

Fifty seven (54.3%) were at risk of malnutrition or were malnourished, 22 (21.0%) had lost weight in the previous year and only 13 (12.4%) were obese [Body Mass Index (BMI) (mean = 27.0 SD = 5.11, median 27, IQR 7.45)]. Urinary incontinence (UI) was reported in 36 (34.6%) residents and faecal incontinence in 36 (34.6%).

In terms of functional capacity, according to the Barthel test, 5 (4.8%) were independent, 11 (10.6%) were slightly dependent, 40 (38.5%) moderately dependent and 48 (46.1%) totally dependent. Forty-six (44.2%) had a gait speed of less than 0.8 m/s, only 3 (2.9%) had a higher Gait Speed Test (SPPB) and 51 (52.9%) were not evaluated. Seventy-eight (75.0%) had cognitive impairment on the Pfeiffer Scale: 25 (24.0%) had moderate cognitive impairment and 53 (51.0%) had severe cognitive impairment.

The residents had an average wake time of 10.7 (SD = 1.16) hours. During waking hours, residents spent a mean of 9.0 (SD = 1.64) hours in SB (sitting or reclining). Although the residents spent 84.2% (SD = 16.85) of their total waking time in SB, in these waking hours the residents also spent 1.6 (SD = 1.91) hours in an upright position (standing or stepping), walking an average of 1345 (SD = 2417.40) steps per day. They transitioned from sitting to standing an average of 18.2 (SD = 18.28) times across a 24 h period (Table 1).

**Table 1:** Descriptive analysis of the sample of institutionalized older adults (*n* = 104)

Variables	Frequency (%) / mean (standard deviation)	Median / IQR
**Age**	**84.6 (SD = 7.8)**	**86 (11)**
**Sex**		
Women	88 (84.6%)	
Men	16 (15.4%)	
**NH Type**		
State Subsidized places	86 (82.7%)	
Private	18 (17.3%)	
**Chronic Disease**	**5 (SD = 2.4)**	**5 (3)**
Hypertension	62 (32.5%)	
Dementia	62 (32.5%)	
Cardiac pathology	43 (22.5%)	
Depression	32 (16.8%)	
Diabetes mellitus II	32 (16.8%)	
Kidney failure	26 (13.6%)	
CVA (cerebral stroke)	21 (11.0%)	
Pulmonary pathology	20 (10.5%)	
Mental pathology	20 (10.5%)	
Cancer	18 (9.6%)	
Parkinson	14 (7.3%)	
**Smoke**		
Yes	6 (3.1%)	
No	98 (96.9%)	
**Alcohol**		
Yes	9 (4.7%)	
No	95 (95.3%)	
**Drugs**^**a**^	**6.9 (SD = 3.8)**	**6.5 (5)**
Group N	98 (55.1%)	
Group A	67 (36.6%)	
Group C	17 (13.1%)	
Group B	44 (23.1%)	
Group R	56 (33.1%)	
Group H	16 (8.4%)	
Group G	7 (4.2%)	
Group M	7 (4.2%)	
Group S	5 (2.7%)	
Group J	4 (2.4%)	
Group D	4 (2.4%)	
Group L	1 (0.7%)	
Group V	1 (0.7%)	
**Nutritional Status**		
Good Nutrition	47 (45.7%)	
Malnutrition or malnourished	57 (54.3%)	
**Weight loss**		
Yes	22 (21.0%)	
No	82 (79%)	
**Obesity**		
Yes	13 (12.4%)	
No	91 (87.6%)	
**Urinary incontinence**		
Yes	36 (34.6%)	
No	68 (65.4%)	
**Faecal incontinence**		
Yes	36 (34.6%)	
No	68 (65.4%)	
**Functional Capacity (Barthel)**		
Independence	5 (4.8%)	
Slight dependence	11 (10.6%)	
Moderated dependence	40 (38.5%)	
Total dependence	48 (46.1%)	
**Cognition (Pfeiffer Scale)**		
No cognitive impairment	16 (15.4%)	
Mild cognitive impairment	10 (9.6%)	
Moderate cognitive impairment	25 (24.0%)	
Sever cognitive impairment	53 (51.0%)	
**Gait speed (SPPB)**		
−/=0.8 m/s	46 (44.2%)	
+ 0.8 m/s	3 (2.9%)	
Not evaluated	51 (52.9%)	
**Sedentary Behavior**		
Average wake time	10.7 (SD = 1.16)	
SB (sitting or reclining) in hours	9.0 (SD = 1.64)	
Total waking time in SB (%)	84.2% (SD = 16.85)	
Hours in upright position (standing or stepping)	1.6 (SD = 1.91)	
Steps per day	1345 (SD = 2417.40)	
Sitting to standing transitions	18.2 (SD = 18.28)	

^a^ Drugs: N (Nervous System), A (Alimentary tract and metabolism), C (Cardiovascular system), B (Blood and blood forming organs), R (Respiratory System), H (Systemic hormonal preparations, excl. Sex hormones and insulins), G (Genito urinary System/sex hormones), M (Musculo-skeletal system), S (Ophthalmologicals), J (Antiinfectives), D (Dermatologicals), L (Antineoplastic agents) and V (Immunomodulating agents)

*IQR* Interquartile range

### Prevalence and severity of sarcopenia in residents living in NHs

**Fig. 2:**
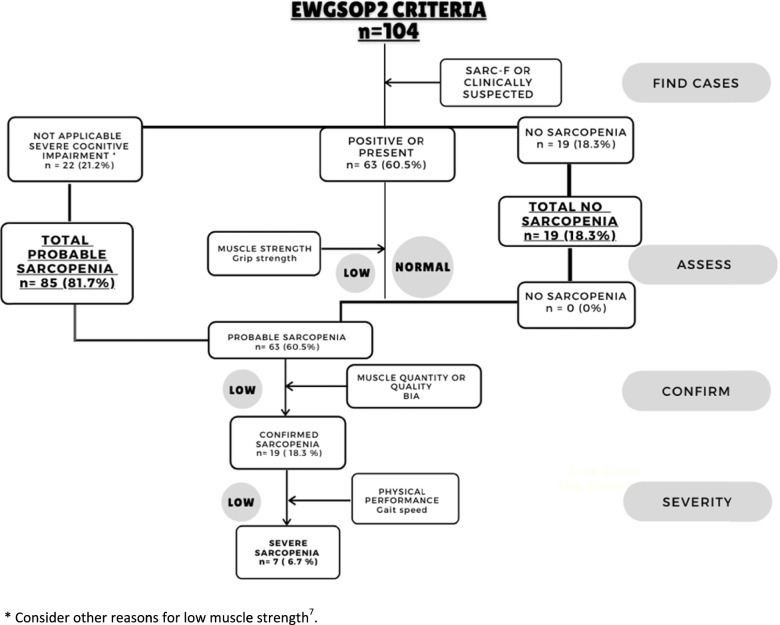
Classification of sarcopenia among institutionalized older people, according to the EWGSOP2 algorithm for case-finding, making diagnosis and quantifying severity

Eighty-five (81.7%) residents were categorized with total probable sarcopenia: 22 (21.1%) of these residents were given this diagnosis as they had severe cognitive impairment so could not perform physical tests and 63 (60.5%) were given this diagnosis for having low muscle strength values. Of those with probable sarcopenia, 19 (18.3%) were diagnosed with confirmed sarcopenia because of low muscle mass. Finally, 7 (6.7%) individuals were diagnosed with severe sarcopenia because of the inability to walk or slow walking speed. Nineteen (18.2%) NH residents had no sarcopenia using the EWGSOP2 criteria (Fig. 2).

### Associated factors of sarcopenia in residents living in NHs

In the bivariate analysis, total probable sarcopenia was significantly associated with nutritional status, obesity, UI and % time in SB. In the multivariate analysis, the variables associated with probable sarcopenia were obesity and UI. The significance of the model with the Hosmer Lemeshow test was *p* = 0.231 (Table 2).

**Table 2:**
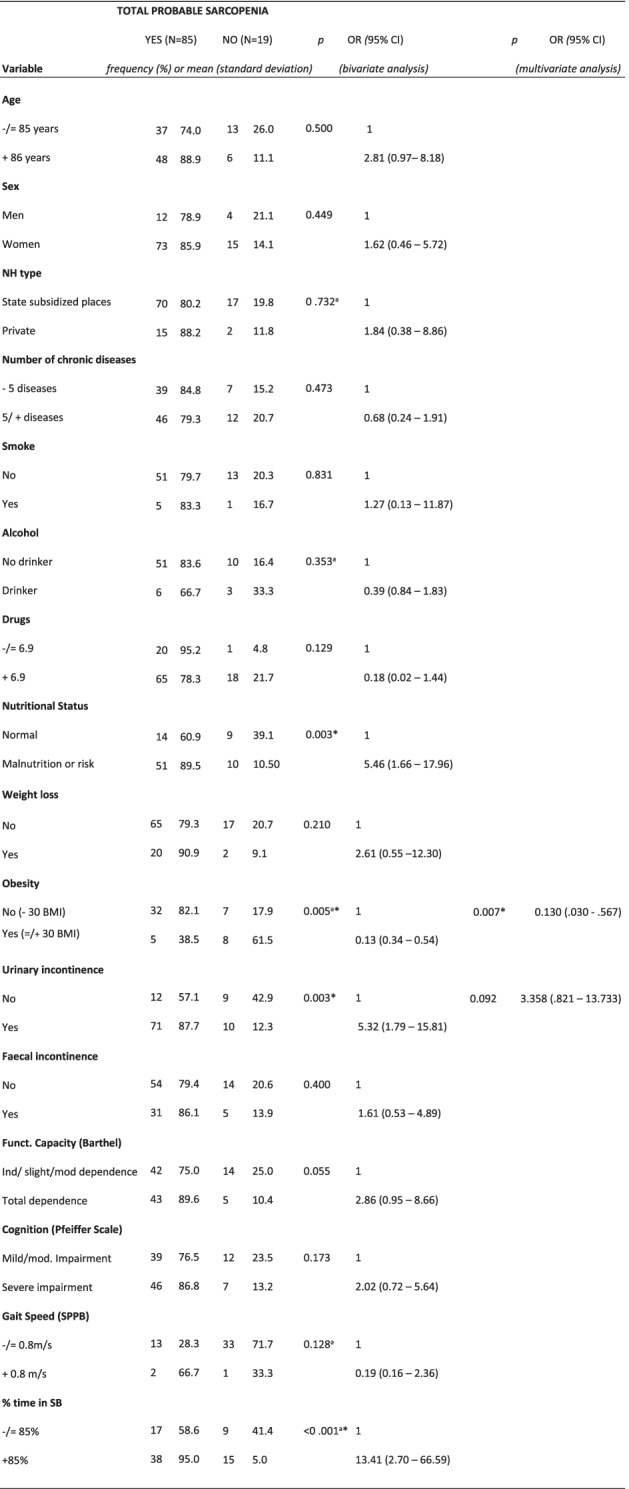
Bivariate and multivariate analysis showing factors associated with total probable sarcopenia according to the EWGSOP2

*OR* Odds Ratio, *CI* Confidence interval, *NH* Nursing Home, *BMI* Body mass index, *SPPB* Short Physical Performance Battery, *SB* Sedentary behavior.

*Key:*^a^Fisher’s exact text.

*Statistically significant (< 0.05)

Probable sarcopenia showed significant associations with nutritional status, obesity, UI and % time in SB. In the multivariate analysis, the Hosmer Lemeshow test was *p* = 0.209 (Table 3).

**Table 3:**
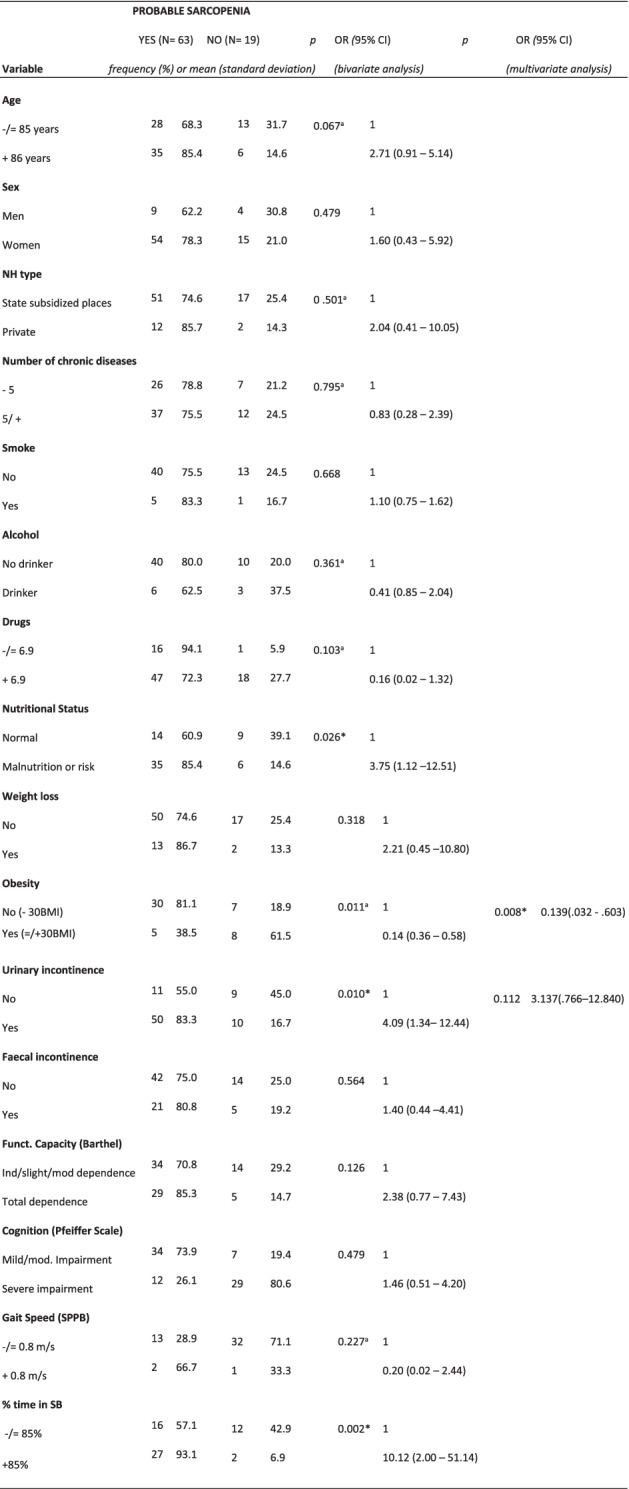
Bivariate and multivariate analysis showing factors associated with probable sarcopenia according to the EWGSOP2

*OR* Odds Ratio, *CI* Confidence interval, *NH* Nursing Home, *BMI* Body mass index, *SPPB* Short Physical Performance Battery, *SB* Sedentary behavior.

Key: ^a^Fisher’s exact text.

* Statistically significant (< 0.05)

Confirmed sarcopenia showed significant associations with obesity and % time in SB. In the multivariate analysis, the Hosmer Lemeshow test was *p* = 1.000 (Table 4).

**Table 4:**
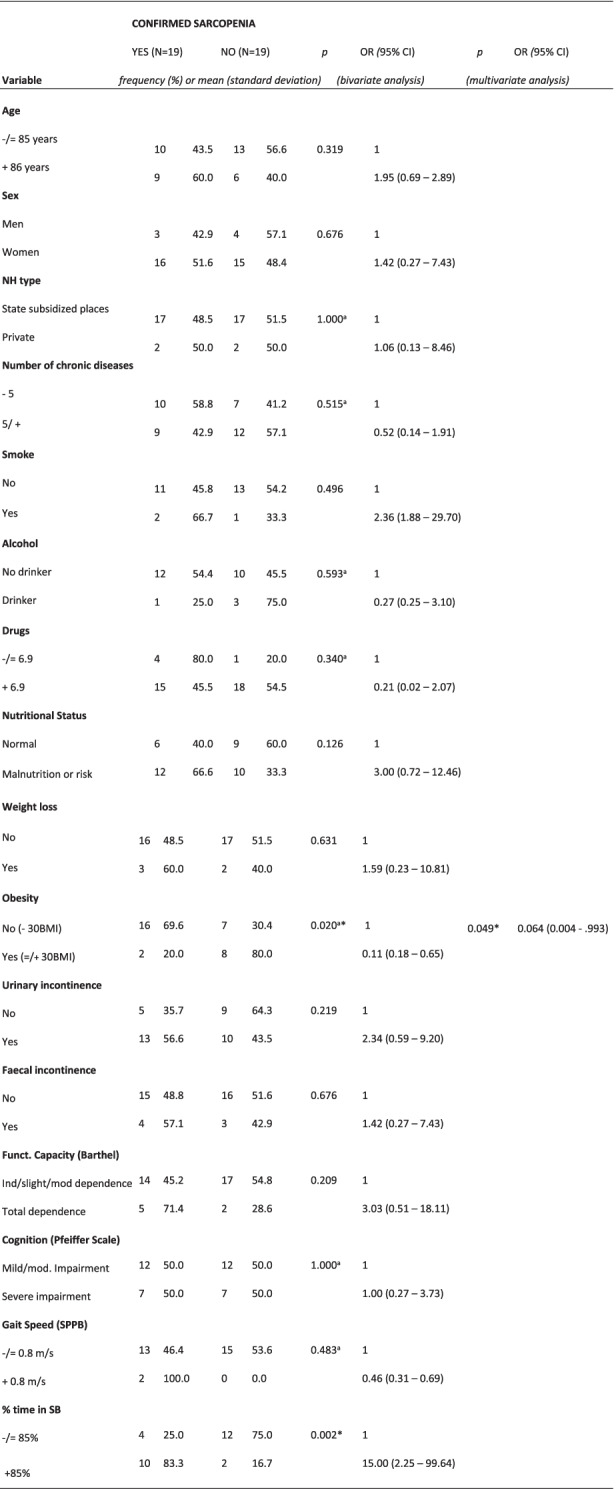
Bivariate and multivariate analysis of factors associated factors with confirmed sarcopenia according to the EWGSOP2.

*OR* Odds Ratio, *CI* Confidence interval, *NH* Nursing Home, *BMI* Body mass index, *SPPB* Short Physical Performance Battery, *SB* Sedentary behavior.

*Key:*^a^ Fisher’s exact text.

* Statistically significant (< 0.05)

Severe sarcopenia only showed a significant association with obesity and % time in SB (Table 5).

**Table 5:**
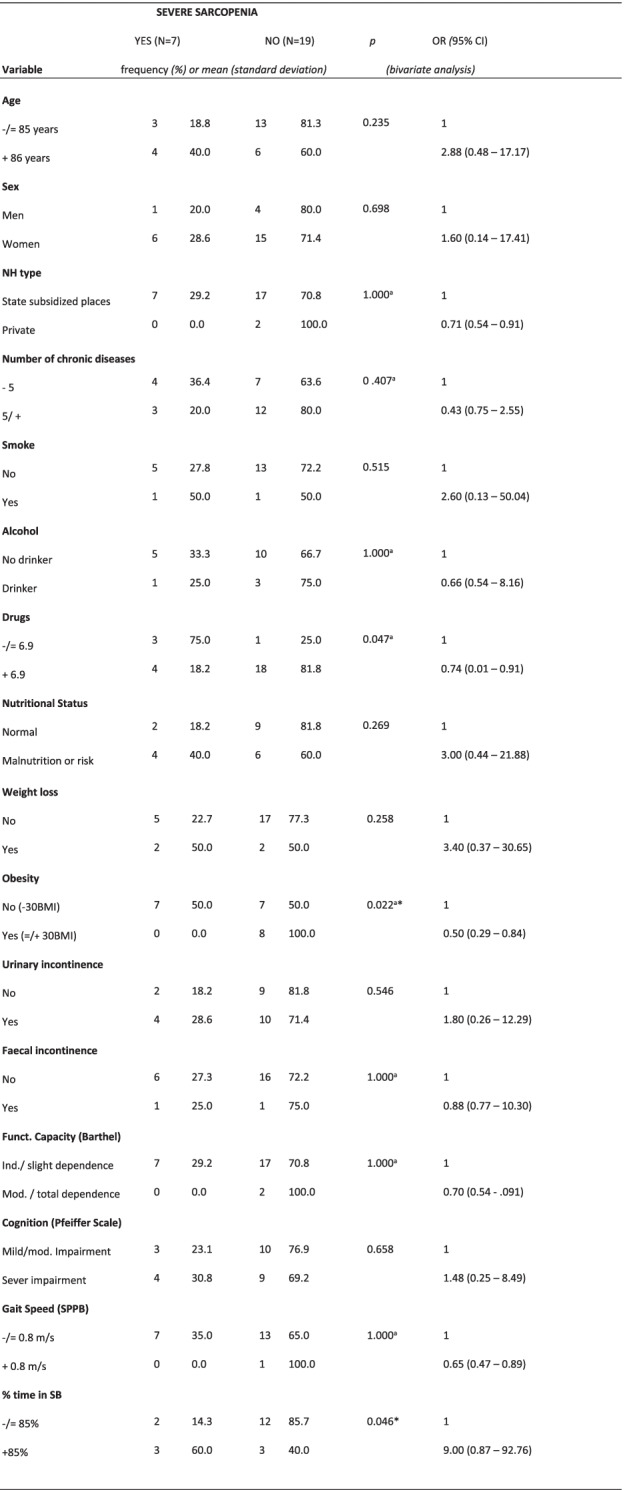
Bivariate analysis of factors associated with severe sarcopenia according to the EWGSOP2

*OR* Odds Ratio, *CI* Confidence interval, *NH* Nursing Home, *BMI* Body mass index, *SPPB* Short Physical Performance Battery, *SB* Sedentary behavior.

*Key:* a Fisher’s exact text.

* Statistically significant (< 0.05)

For the multivariate analysis (Tables 2, 3, 4 and 5), obesity (as a nutritional variable or variable of nutritional status) was combined with UI or % time in SB. Obesity was negatively associated with total probable sarcopenia, probable sarcopenia and confirmed sarcopenia, independent of UI. Obesity and % time in SB showed a significant association with confirmed sarcopenia. The multivariate analysis was not possible for the last level (severe sarcopenia), due to the small sample size.

## Discussion

The main objective of this study was to identify the prevalence of sarcopenia in older people living in NHs. The results showed a high prevalence of sarcopenia, 81.7% having the presence of some category of sarcopenia. Of those categorized as sarcopenic, 60.5% had probable sarcopenia, 18.3% had confirmed sarcopenia, and 6.7% had severe sarcopenia according to the new EWGSOP2 criteria. A recent systematic review showed that the prevalence of sarcopenia in their included studies varied from 22 to 85.4% [5], therefore our results are into the high part of this range, but multiple criteria for categorizing sarcopenia were used. Other cross-sectional studies [12, 32] reported the prevalence of sarcopenia in NHs according to the new EWGSOP2 criteria, suggesting a high frequency of some category of sarcopenia, ranging from 73 to 91%, more in line with our findings. However, within these studies the severity of sarcopenia was higher than we find in our study, probably because the participants were older than ours [12].

A fifth of residents in our study had severe cognitive impairment, meaning some of the tests to confirm sarcopenia risk, diagnosis or severity could not be performed. The literature also suggests a high prevalence of people with cognitive impairment and/or sarcopenia (from different criteria) in NHs [5, 16]. In our study we observed that an optimal cognitive status is necessary to determine the degree of severity of sarcopenia using the tests proposed by the EWGSOP2. However, we included all residents within our study, using proxy criteria to determine sarcopenic status.

We found that nutritional variables such as malnutrition and obesity, UI and % time in SB were significantly associated sarcopenia risk factors in NH residents. Furthermore, multivariate analysis showed a negative association of obesity with total probable sarcopenia, with probable sarcopenia and with confirmed sarcopenia; and a positive association of % time in SB with confirmed sarcopenia.

Our results indicate that obesity acts as a protective factor for sarcopenia, with obese subjects having a lower risk of being categorized as sarcopenic. Faxén-Irving et al. [12], who applied the EWGSOP2 criteria, did not include overweight and obese residents in their study and this may explain why they report a higher prevalence of severe sarcopenia than seen in our study. Other literature confirms an association of sarcopenia with obesity. Halil et al. [34] also concluded that sarcopenia was inversely associated with BMI. During aging, involuntary weight loss (anorexia of aging) is an indicator of frailty and may accelerate the process of muscle wasting. Those who do not experience age-related weight loss may be better able to maintain muscle mass and thus muscle strength [34]. Those who do not maintain weight and show signs of malnutrition are more likely to be categorized as sarcopenic in our study. Pereira et al. confirmed our findings, identifying that two out of three institutionalized older adults had malnutrition and sarcopenia [35]. Malnutrition leads to lower muscle strength and less physical activity [36], which is reflected in this study by the low scores in the hand-grip muscle strength and in the Gait Speed, and reinforced by the high % time in SB [36], which also was identified as an associated factor with sarcopenia.

Two studies report that that higher levels of SB were found to be associated with higher levels of sarcopenia [36, 37]. Physical inactivity contributes to development of sarcopenia, whether due to disease-related immobility or disability, or to a sedentary lifestyle, which has been shown to be a risk factor for muscle weakness that in turn, results in reduced activity levels, loss of muscle mass, and muscle strength [38]. The association of UI with sarcopenia seen in our study is confirmed by a recent study [39] which concluded that the prevalence of sarcopenia in women with pelvic floor dysfunction was high, revealing that UI is strongly associated with musculoskeletal conditions and impaired mobility function in older adults.

Our results confirm the clinical significance of interventions that include adequate nutritional support and physical exercise to improve the adverse outcomes of sarcopenia in older people living in NHs. Therefore, diagnosis of sarcopenia is very important in residential settings [40].

The main limitation of the study lies in the relatively small sample included in the study due to the onset of the COVID-19 pandemic that prevented further data collection. However, we were able to recruit more than our sample size calculations recommended. Another important barrier was the high prevalence of people with cognitive impairment in NHs, meaning difficulties in performing the EWGSOP2 tests. For this reason, further studies are needed to verify the usefulness of the new diagnostic criteria in institutionalized older adults. However, we were able to use proxy measures for categorizing those with cognitive impairment not able to take part in the functional tests, allowing us to include all residents in this setting.

The strength of this work is the involvement of all residents and the use of the most recent consensus based criteria to diagnose sarcopenia, the new EWGSOP2 algorithm, in order to verify prevalence, severity, and associated factors in NH residents. Furthermore, these associated factors were analyzed considering a wide range of variables to assess health, based on the biopsychosocial model of health. It is now important that we strive to deliver evidence-based interventions in these settings to mitigate sarcopenia and its associated health outcomes.

## Conclusions

According the EWGSOP2 criteria, a high prevalence of sarcopenia was found in this sample of institutionalized older people, ranging from 6.7 to 81.7% depending on the category of sarcopenia. Malnutrition, urinary incontinence and sedentary behaviour were associated with sarcopenia whilst obesity represented a protective factor in this population. In terms of implications for clinical practice, evidence-based interventions including physical activity and nutritional diet will be essential to decrease sarcopenia prevalence.

## Data Availability

The datasets used and/or analyzed during the current study are available from the corresponding author on reasonable request.

## Abbreviations

EWGSOP2: European Working Group on Sarcopenia in Older People
NH: Nursing Home
STROBE: Strengthening the Reporting of Observational Studies in Epidemiology
BIA: Bioimpedance Analysis
SMM: Skeletal Muscle Mass
Ht: Height in centimeters
R: Resistance in Ohms
SPPB: Short Physical Performance Battery
MNA: Mini Nutritional Assessment
MDS: Minimum Data Set
ICC: Interclass Correlation Coefficient
OR: Odds Ratio
UI: Urinary Incontinence
BMI: Body Mass Index
SD: Standard Deviation
